# First steps towards the successful surface-based cultivation of human embryonic stem cells in hanging drop systems

**DOI:** 10.1002/elsc.201100213

**Published:** 2012-08-01

**Authors:** Julia C Schulz, Patrick S Stumpf, Alisa Katsen-Globa, Agapios Sachinidis, Jürgen Hescheler, Heiko Zimmermann

**Affiliations:** 1 Fraunhofer Institute for Biomedical EngineeringGermany; 2 Center of Physiology and Pathophysiology, Institute of Neurophysiology, University of CologneGermany; 3 Chair of Molecular and Cellular Biotechnology/Nanotechnology, Saarland UniversitySaarbrücken, Germany

**Keywords:** Hanging drop, High-throughput screening, hESCs, Microcarrier

## Abstract

Miniaturization and parallelization of cell culture procedures are in focus of research in order to develop test platforms with low material consumption and increased standardization for toxicity and drug screenings. The cultivation in hanging drops (HDs) is a convenient and versatile tool for biological applications and represents an interesting model system for the screening applications due to its uniform shape, the advantageous gas supply, and the small volume. However, its application has so far been limited to non‐adherent and aggregate forming cells. Here, we describe for the first time the proof-of-principle regarding the adherent cultivation of human embryonic stem cells in HD. For this microcarriers were added to the droplet as dynamic cultivation surfaces resulting in a maintained pluripotency and proliferation capacity for 10 days. This enables the HD technique to be extended to the cultivation of adherence-dependent stem cells. Also, the possible automation of this method by implementation of liquid handling systems opens new possibilities for miniaturized screenings, the improvement of cultivation and differentiation conditions, and toxicity and drug development.

Although stem cell research is one of the most promising areas in biomedicine, efficient expansion and differentiation of human embryonic stem cells (hESCs) have yet to be optimized. The identification of small molecules promoting specific differentiation of hESCs is ineffective, time consuming, and labor intensive with the current manual cell culture techniques. Furthermore, effective culture techniques allowing investigation of the potential synergic effects of two or more compounds on propagation and differentiation of hESCs need to be developed. Consequently, systematic study of culture conditions, as well as optimization and standardization of culture, and differentiation protocols is needed both for potential hESC-based therapeutical purposes and for basic research. No currently available culture technique is affordable under the high demands of multifactorial human stem cell cultivation for clinical use.

Here, we describe a new, miniaturized, and parallelized cell cultivation and differentiation system with great potential for automation and screening applications. The hanging drop (HD) technique has a long and versatile history in biomedicine. It was first used in 1907 by Harrison et al. [1] for the cultivation of nerve tissue and subsequent applications range from developmental biology [2,3] to tissue engineering 4, and stem cell differentiation [5]. For these applications, usually 20μL drops of cell suspension are placed on the inner surface of a lid that is then inverted and positioned on the corresponding cell culture dish containing 10 mL buffer solution in order to minimize evaporation. The small compartment size of the drop permits compact but statistically significant experimental layouts, makes low demands on biological material and cell culture components and, with innovative automation approaches [6], is compatible with microfluidic high-throughput screenings. First innovative automation approaches already take advantage of these features [7]. The small size also offers efficient gas exchange, due to the proximity of the liquid–gas interface to the cells, an adequate diffusive dispersion of medium components, and a simplified manipulation of cells without enzymatic treatment. There are already ready-to-use protocols for the differentiation of stem cells [8,9] and the expansion of adherence-independent cell lines has also been reported [10]. To enable the adaption of 2D-based standard protocols for adherent cells to this 3D-system, microcarriers have been added to the drop, providing a cultivation surface for the cells. Cell cultivation on microcarriers has been previously reported in bioreactor culture systems for hESCs [11,12] even under defined culture conditions [13] promising also an improvement of the HD system.

The possible modular supplementation of the adhesion surface is beneficial for screening and for the definition of important cultivation parameters especially in context of the unique, in vivo stem cell niche [14].

Here, the cultivation of H9 hESCs in HD without feeder cells was compared to feeder-dependent standard conditions in 2D described in previous studies [15,16]. For further comparison, hESC culture on six-well culture plates (Nunc, Roskilde, Denmark) coated with human extracellular matrix (MaxGel®, Sigma-Aldrich, Steinheim, Germany, 1:100 in DMEM) for 24 h at 37°C without feeder cells was used in combination with conditioned medium. This medium was preincubated with primary mouse embryo fibroblasts (PMEF; Millipore, Schwalbach, Germany) for 24 h prior to application and was additionally supplemented with bFGF (Invitrogen, Karlsruhe, Germany) to a final concentration of 10 ng/ml, in order to culture the cells in the self-renewal state.

Cytodex 3 microcarriers were coated with human extracellular matrix (ECM; 1:100 in DMEM) for 24 h at 37°C prior to use. After optimizing the microcarrier concentration in the HD with adult stem cells (data not shown), 10 μL drops of conditioned medium with an excess amount of approximately 40 carriers were placed on the inner surface of a lid belonging to a cell culture dish. Analysis of microcarrier distribution per drop showed that 23.25% of the drops contained the applied 30–50 microcarriers, whereas 46.51% of the drops contained more than 70 microcarriers (Fig. 1A). This result guarantees sufficient cultivation surface for an unlimited proliferation of cells, although improvements for a more homogeneous microcarrier distribution are necessary. hESC colonies were manually dissected in fragments of 50–100 μm length using a metal needle designed to prepare samples for scanning electron microscopy. One aggregate was added to each drop to a final volume of 20μL, the lid was inverted and placed on the cell culture dish containing buffer solution. The hESC colonies were kept for 10 days in HD culture with a medium exchange every other day. Therefore, the lid with the drops was inverted and half of the medium was carefully renewed.

**Figure 1:**
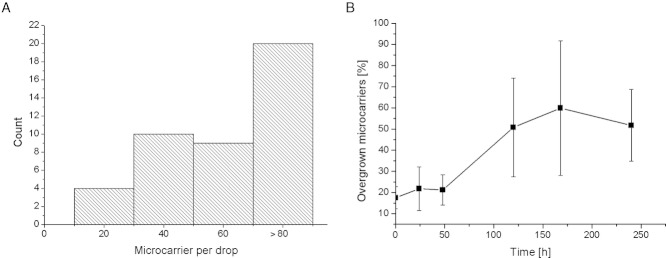
Distribution of microcarriers per drop (A) and proliferation of hESCs over time (B). (A) The number of microcarriers per drop was counted based on a starting concentration of 2 microcarriers per μl and the distribution was figured as histogramm. (B) The amount of overgrown microcarriers was determined manually for 10 days as evidence for cell proliferation by analyzing the corresponding bright field images; *n*≥5 drops per time point.

The most important parameters for successful hESC culture are proliferation and the reliable maintenance of pluripotency in HD culture for prolonged time. Proliferation of colonies is not easy to monitor quantitatively due to the diverging cell numbers of the manually dissected cell colonies. Nevertheless, the amount of overgrown microcarriers was determined over time as evidence for cell proliferation by analyzing the bright field images (Fig. 1B). For better quantification, drops with about 30 microcarriers and one hESC aggregate have been used. Due to the still existing surplus of microcarriers, there should be no limitation of the proliferation potential of hESCs. Microscopical analysis showed that cells proliferated manifold, surrounded the Cytodex 3 microcarriers coated with human ECM as a monolayer and colonized further beads that were then incorporated inside the growing cell-microcarrier aggregate (Fig. 2A). Analysis of the amount of overgrown microcarrier resulted in an increase from 17.49 ± 5.26% on day 0 to 21.82 ± 10.28% after 24 h and to 59.94 ± 31.83% on day 7. After that, proliferation stagnated on day 10 (51.70 ± 16.97%) probably due to limitations in nutrient and gas supply.

**Figure 2:**
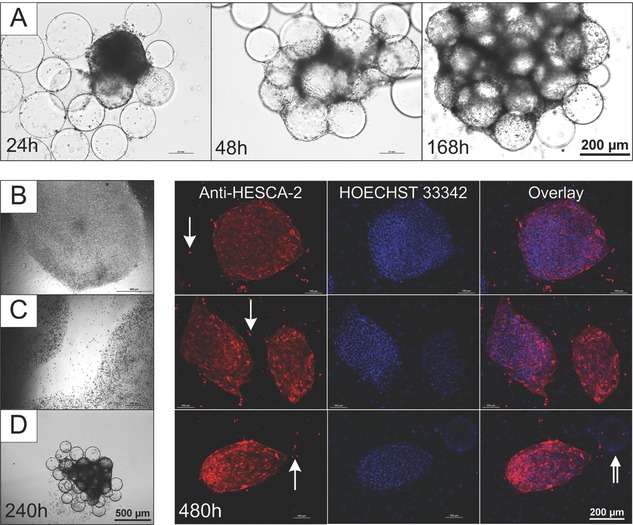
Growth and stemness of human embryonic stem cells (hESCs) in hanging drop (HD) culture without feeder cells, maintained over 10 days. (A) Inoculated hESCs colonized further microcarriers as they grow as monolayer around the beads during HD cultivation. (B-D) Comparison of hESC stemness under various culture conditions using bright field images on day 10 of cultivation in 2D- and 3D-systems and their corresponding immunocytochemical staining against the stemness antigen HESCA-2 with a counterstaining for nuclei and overlay after two subsequent passages. (B) Standard culture of hESCs on feeder cells. (C) Culture with conditioned medium on culture dishes coated with human human extracellular matrix (ECM) without feeder cells. (D) HD culture with conditioned medium on Cytodex 3 microcarriers coated with human ECM as adhesion surface without feeder cells. Arrows indicate single cells outside the hESC colonies which are positively stained for stemness; double arrow shows a residual microcarrier after replating the cells in 2D culture.

After cultivation, all colonies were completely detached from the microcarriers using 0.1% collagenase (Sigma-Aldrich) solution and were replated under standard culture conditions on mitotically inactivated feeder cells for investigating the long-term effects on cell characteristics. After at least 5 days, cells were fixed for immunocytochemistry using 2% glutaraldehyde in 0.15 M sodium cacodylate buffer, stained with a specific stemness antibody (anti-HESCA-2, 1:100, Millipore) and counterstained with 10 mg/L of the nucleic acid dye HOECHST 33342 (Invitrogen). Positive immunocytochemical staining of all samples after repassaging to standard culture conditions (fluorescence images in Fig. 2B-D) provided the first evidence for maintenance of pluripotency during HD cultivation. No difference in stemness could be detected between the different conditions, though minor spontaneous differentiation occurred in all samples, a process commonly detectable in every in vitro cultivation method [16]. Nevertheless, future studies have to focus on advanced investigations of stemness and proliferation of hESCs using standard assays such as karyotyping and teratoma formation [17].

In summary, we have provided the proof-of-principle regarding the extended cultivation of strictly adherence-dependent hESCs in HD without feeder cells. Fundamental culture conditions were established that can be further optimized in comprehensive screenings. For high-throughput applications, robotic liquid handling systems can remove much of the manual labor from HD cultivation. This is currently tested with a range of stem cell lines. The manipulation of drops was enabled via a whole from above, so that automated drop generation, medium exchange, and drop harvest has already been successfully demonstrated with no adverse effects on cell viability (data not shown). The modified HD method presented here is highly amenable to parallelization and automation. Meaning that rapid, cost-effective, and highly precise investigation of culture conditions will be possible in future, particularly by implementing liquid handling systems and innovative readout-systems, e.g. the Opera® from PerkinElmer. As every single droplet represents a small and separate bioreactor, this methodology enables automated investigations of thousands of different conditions in parallel, thus facilitating the finding of optimal culture and differentiation protocols required for hESC applications in stem cell therapy and basic research. But nevertheless, for reproducible and standardized applications future optimization is required due to the huge standard deviations regarding the number of microcarriers overgrown with hESCs and regarding the inhomogeneous distribution of microcarriers per droplet.

## Abbreviations

hESCs: human embryonic stem cells
HD: hanging drop
